# Supplementary material for: Is SORL1 a common genetic target across neurodegenerative diseases?: A multi-ancestry biobank scale assessment
Source: medRxiv. 2026 Feb 2:2026.01.24.26344530. Originally published 2026 Jan 30. Preprint. [Version 2] doi: 10.64898/2026.01.24.26344530 (PMC12870714; doi:10.64898/2026.01.24.26344530)
Supplement: Supplement 1 [file media-1.pdf]

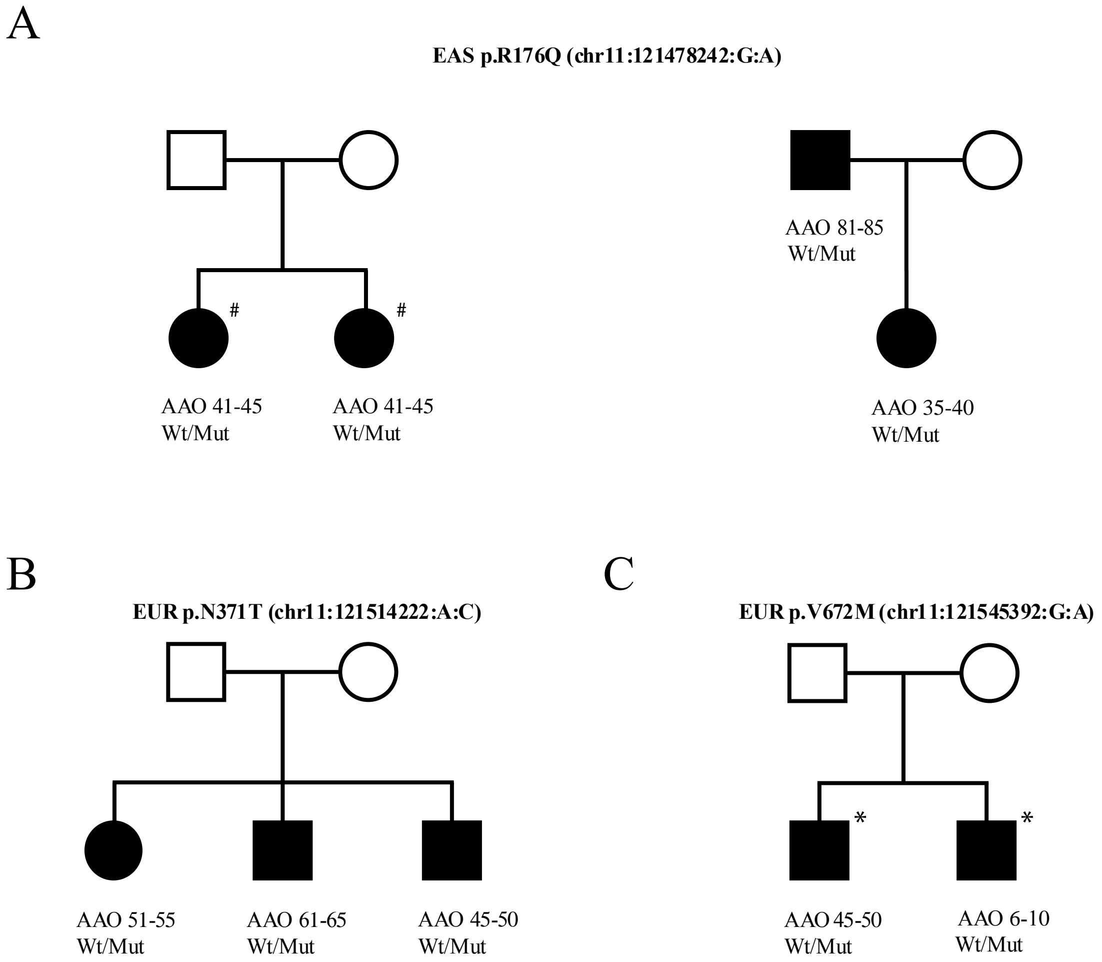
A
EAS p.R176Q (chr11:121478242:G:A)
#
#
AAO 41-45
Wt/Mut
AAO 41-45
Wt/Mut
AAO 81-85
Wt/Mut
AAO 35-40
Wt/Mut
B
EUR p.N371T (chr11:121514222:A:C)
AAO 51-55
Wt/Mut
AAO 61-65
Wt/Mut
AAO 45-50
Wt/Mut
C
EUR p.V672M (chr11:121545392:G:A)
*
*
AAO 45-50
Wt/Mut
AAO 6-10
Wt/Mut
