# Supplementary material for: Is SORL1 a common genetic target across neurodegenerative diseases?: A multi-ancestry biobank scale assessment
Source: medRxiv. 2026 Feb 2:2026.01.24.26344530. Originally published 2026 Jan 30. Preprint. [Version 2] doi: 10.64898/2026.01.24.26344530 (PMC12870714; doi:10.64898/2026.01.24.26344530)

medRxiv preprint doi: https://doi.org/10.64898/2026.01.24.26344530; this version posted February 2, 2026. The copyright holder for this preprint
(which was not certified by peer review) is the author/funder, who has granted medRxiv a license to display the preprint in perpetuity.
This article is a US Government work. It is not subject to copyright under 17 USC 105 and is also made available
for use under a CC0 license.

Supplementary Figure 1- *SORL1* candidate-variant carrier pedigrees. (A) Two East Asian (EAS) families carrying *SORL1* p.R176Q (11:121478242:G:A). (B) European (EUR) family carrying *SORL1* p.N371T (11:121514222:A:C). (C) European (EUR) family carrying *SORL1* p.V672M (11:121545392:G:A). Squares indicate males and circles indicate females; filled symbols denote individuals affected with PD, unfilled symbols indicate samples without genetic data available. Age at onset (AAO) is shown when available. Genotype is shown as Wt/Mut (heterozygous) for the respective *SORL1* variant. # indicates individual carriers of the PD associated mutation p.L483P in *GBA1*. * indicates individual carriers of the PD associated mutation p.G192R in *RAB39B*.

medRxiv preprint doi: https://doi.org/10.64898/2026.01.24.26344530; this version posted February 2, 2026. The copyright holder for this preprint (which was not certified by peer review) is the author/funder, who has granted medRxiv a license to display the preprint in perpetuity.
This article is a US Government work. It is not subject to copyright under 17 USC 105 and is also made available

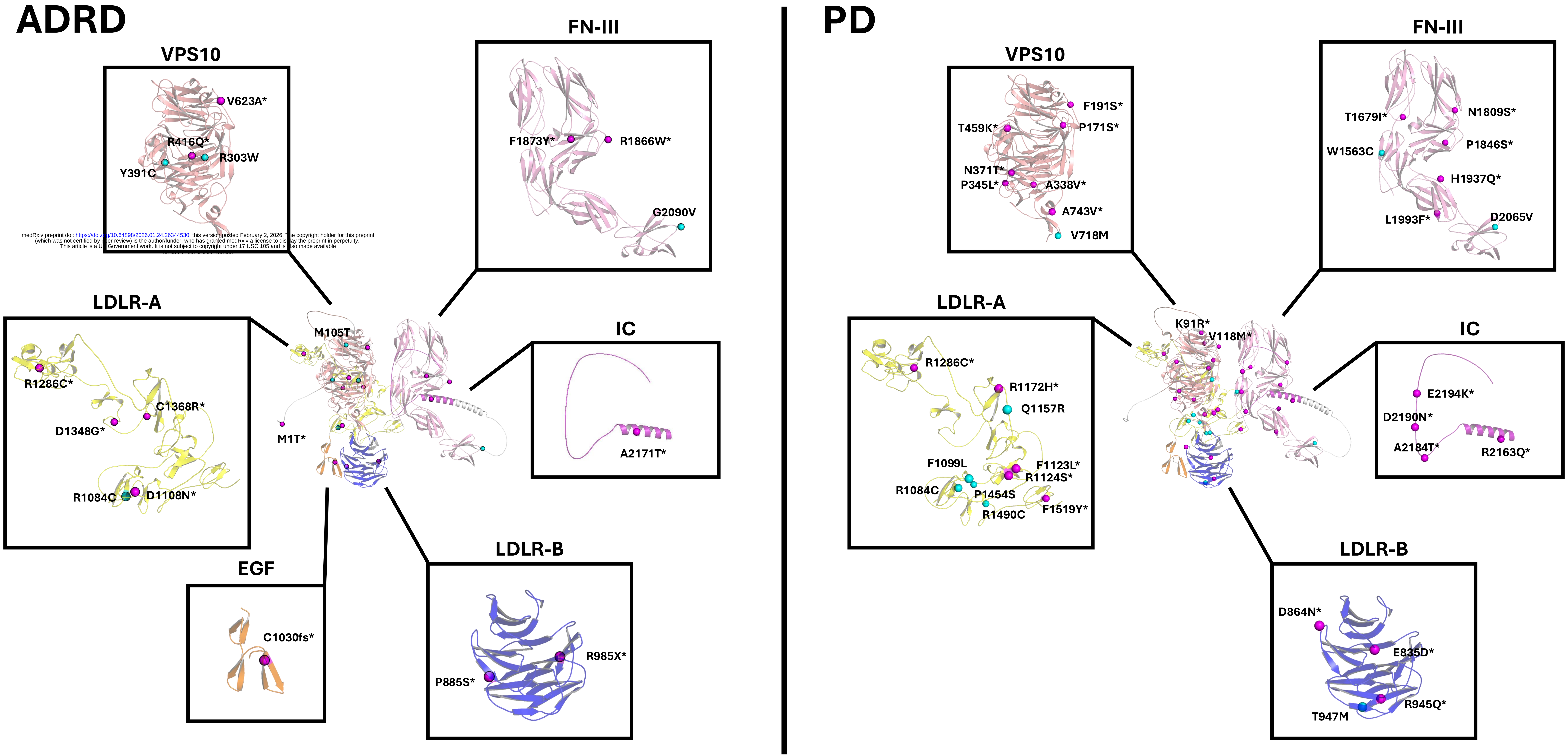

Supplement: 9 [file NIHPP2026.01.24.26344530v2-supplement-9.pdf]
